# The experience of meaningful rehabilitation as perceived by people with chronic pain: A phenomenological study

**DOI:** 10.3233/WOR-220119

**Published:** 2023-06-13

**Authors:** Katrina J. Liddiard, Annette J. Raynor, Helen DeJong, Cary A. Brown

**Affiliations:** a School of Medical and Health Sciences, Edith Cowan University, Joondalup, WA, Australia; b Perth Scar and Pain Clinic, Mount Pleasant, WA, Australia; c Department of Occupational Therapy, Faculty of Rehabilitation Medicine, University of Alberta, Edmonton, AB, Canada

**Keywords:** Partner, power, pain management, therapeutic alliance, client-centered therapy

## Abstract

**BACKGROUND:**

People with chronic pain may seek rehabilitation to reduce pain and restore productivity and valued roles. Theoretically, a biopsychosocial approach makes rehabilitation more meaningful, however, the limited research on meaningful rehabilitation predominantly describes the perspective of therapists and researchers. The client’s perspective of meaningfulness in rehabilitation is lacking.

**OBJECTIVE:**

To investigate the experience of meaningfulness in rehabilitation from the perspective of people with chronic pain.

**METHODS:**

Qualitative, semi-structured interviews were conducted with Australian adults who had chronic pain and recent experience of occupational therapy or physiotherapy. Sampling continued until thematic saturation occurred. Transcripts were coded and analyzed using theory-driven and data-driven thematic analysis.

**RESULTS:**

Ten participants (four males; six females) were interviewed. Pain histories ranged from nine months to 20+ years, with conditions such as fibromyalgia or trauma. Three themes from a prior concept analysis were upheld, and a further three data-driven themes emerged. Results indicate that people with chronic pain seek a “*genuine connection*”; from a therapist who is “*credible*”; and can become a “*guiding partner*”, and they find rehabilitation meaningful when it holds “*personal value*”; is “*self-defined*”; and relevant to their sense of “*self-identity*”.

**CONCLUSIONS:**

The *genuine connection* and *guiding partnership* with a *credible* therapist, that is sought by people with chronic pain, may be at odds with aspects of contemporary rehabilitation. Client-defined meaningfulness is an important construct to engage clients in treatment and improve work and other occupational outcomes for people with chronic pain.

## 1 Introduction

Pain is processed by the central nervous system and modulated by cognitive and emotional factors [1]. Chronic pain is a state in which neurobiological, psychological, and social mechanisms interact to amplify and sustain the pain experience [1]. Estimates of chronic pain prevalence vary, however, 3.24 million Australians were believed to be living with chronic pain in 2018 [2], and incidence rates in other parts of the world are similar or higher [3]. Chronic pain can significantly reduce quality of life [2] through occupational, financial, physical, emotional and social impacts; and can reduce a person’s ability to carry out their usual roles and functions within vocational, family and social contexts [4–6]. Specific occupational roles may put people at greater risk of chronic pain conditions [7] and people from marginalized or disadvantaged groups, such as those with severe disability or at socioeconomic disadvantage, may be over-represented and more greatly affected by chronic pain [8, 9]. This is important because therapists, who come from an inherent position of power, may not recognize the hierarchy in the therapist-client relationship [10]. Therapists and clients will not necessarily have a shared world view, and this could lead to faulty assumptions about what will make the rehabilitation encounter meaningful from the client’s perspective.

Current chronic pain research often frames quality of life and well-being as important [11–13], thus moving beyond the singular aim of pain reduction. Rehabilitation is seen as a strategy to restore participation in meaningful or productive life roles and reduce pain whilst improving quality of life [14]. To facilitate clients’ goal of well-being therapists can apply contemporary systems-level practice models to guide their conceptualization of chronic pain as a complex phenomenon which requires a biopsychosocial approach [15, 16]. This comprehensive lens is important because of the complex and evolving conditions which interact to influence, and be influenced by the experience of chronic pain [15, 17, 18].

Specialist pain health professionals support the concept of a biopsychosocial approach as the gold standard for chronic pain management [15, 19], however, generalist health professionals may still be more inclined to retain a biomedical perspective [20]. Even when therapists acknowledge the importance of a biopsychosocial model, they may apply it with a reductionist view more common to a biomedical model [21]. Therapists may compartmentalize the biological, psychological and social aspects rather than considering them as an interactive whole. There is a risk therefore, that the psychological and social factors are addressed alongside the biological, rather than in conjunction with the biological [21]. There is some evidence that the ‘bio’ receives greater attention from health professionals than the ‘psychosocial’ [22]. For example, surveys of occupational therapists’ pain knowledge continue to demonstrate an emphasis on biomedical approaches with concerning gaps in knowledge about psychosocial components [23, 24]. Some physiotherapists may also perceive limitations in their training and education on psychosocial factors [25] or underutilize psychological interventions [26]. Despite emerging evidence that a comprehensive, and integrated, biopsychosocial rehabilitation approach is beneficial [27], there remains an important knowledge gap about what makes chronic pain rehabilitation meaningful from the client perspective.

The client’s experience with rehabilitation can be influenced by the therapist [28, 29]. For example, the alliance between therapist and client, also referred to as therapeutic relationship, can have a positive impact on rehabilitation [30], and clients who perceive therapy to be “goal oriented, meaningful and enjoyable” [31] are also known to have better outcomes. The therapeutic relationship is certainly an important part of contemporary chronic pain rehabilitation [28] and may be one factor in a personally meaningful experience of therapy. Despite this, it is not yet clear what other factors make rehabilitation more, or less meaningful from the client’s perspective.

A recent concept analysis [32] identified that the term ‘meaningfulness’ is used in chronic pain rehabilitation literature in different ways. Notably, the client’s voice is missing from representations of what is meaningful in therapy, and the literature predominantly represents meaningfulness from the perspective of the health professional or researcher [32]. For example, “clinically meaningful” [33] is used to describe a drop in pain score; or “meaningful improvement” [34] to report changes in standardized intervention outcome measures [29, 35]. The definition that emerged from the concept analysis was: “Client-identified meaningfulness describes that which *clients themselves select* as being of *value* and contributes to their *personal sense of identity*” [32] and this definition was used to underpin the current study.

In summary, further research into meaningfulness from the client perspective is required in order to develop best-practice guidelines to advance the efficacy of chronic pain rehabilitation. A better understanding of meaningfulness in rehabilitation may facilitate therapy that increases clients’ motivation to engage. In turn, client engagement in therapy is likely to result in more effective outcomes. The aim of this study was to explore the experience of meaningfulness in rehabilitation from the perspective of people with chronic pain.

## 2 Materials and methods

### 2.1 Study design

The study was performed from a constructivist/interpretivist paradigm. A qualitative phenomenological research design [36] was used to examine the experience of meaningfulness in rehabilitation for people with chronic pain. The first author (KL), who is a clinical and academic occupational therapist with pain management experience, conducted interviews and led the data analysis. The research was conducted as part of the first author’s doctoral studies and participants were made aware of this. The research team also included an academic-researcher with experience as a clinical occupational therapist (CB); and an academic-researcher with specialist experience in exercise science and motor learning and control (AR). To minimize researcher bias the lead researcher kept a reflexive journal, a qualitative research method used to examine the researcher’s own research practices, biases and assumptions and improve confirmability [36, 37]. To maintain rigor the research team carried out frequent in-depth debriefs regarding study design, iterative progress and emergent data [37].

### 2.2 Sample

Australian adults with chronic pain, were recruited during February to October 2020. Purposive sampling was used through social media, posters displayed on university campuses, and email via the primary researcher’s therapist colleagues, specialist pain management practices, and larger occupational therapy and physiotherapy practices. Participants were included if they were over 18 years; reported personal experience with chronic pain, described as pain lasting more than three months [38]; and had experience of physiotherapy or occupational therapy within the past 0-52 weeks. Exclusion criteria included those who were non-English speaking; actively attending rehabilitation at the time; and those with significant cognitive impairment. There was no predetermined sample size, as Braun and Clarke [39] recommend that sampling continues until no new themes emerge from the very detailed narrative data, in a process of ‘thematic saturation’ [39].

### 2.3 Data collection

Participants gave informed consent prior to completing the semi-structured in-depth interviews [36] which were conducted in person or via phone by the lead author, according to participant preference. Each participant was interviewed on a single occasion and interviews lasted between 45 and 75 minutes. A question guide was used and modified through an iterative process of concurrent data collection and analysis [40]. Examples of interview questions include:

*For you personally, what would you say was most meaningful in your rehabilitation?*

*How aware do you think your therapist was, that rehabilitation was/wasn*’*t meaningful for you?*

*In what ways do you think you were able to influence the direction of your rehabilitation to make it more meaningful for you?*

Interviews were recorded using a data recorder and transcribed verbatim, either by an external transcription service or by the first author. To minimize bias, as recommended by Liamputtong [36], the interviewer regularly debriefed with the research team to reflect on her role as researcher rather than clinical occupational therapist.

### 2.4 Data analysis

Aligning with the steps outlined by Braun and Clarke [39], transcripts were first checked against the audio recording and edited for accuracy. Initial codes were generated about the experience of meaningfulness in the rehabilitation encounter from the perspective of each participant. Multiple readings of the transcripts ensured the familiarity required for thematic coding [39]. Adhering to practices that facilitate methodological rigor and dependable findings in qualitative research, a codebook and decision audit trail was maintained as codes were merged, refined and developed into themes [37]. Authors KL, AR & CB reviewed transcripts and textual examples regularly to confirm emerging codes and agree on interpretations [37]. Thematic analysis was carried out using the hybrid method described by Braun and Clarke [39]. This method blended theory-driven analysis, to examine whether the client-perspective had any synergy with themes previously identified by Liddiard et al. [32]; with data-driven analysis to identify any additional themes. Themes were reviewed across the entire data set to generate a thematic map and to increase rigor [39]. Themes were then refined, named and given clear definitions for final analysis. To increase dependability the researcher moved repeatedly between coded extracts, the full data set, and themes [37]. Member checking was carried out, and each participant was emailed their own transcript and a short summary of findings for any comments or corrections they wanted to make [36]. Finally, to reduce the risk of coding bias [37] a coding audit of 10% of the data across all transcripts was carried out by the third author (HDJ), who had not been involved in any previous analysis and/or coding discussion. Audit findings were discussed with the first author and 92% agreement was reached.

## 3 Results

A total of ten participants (six women and four men), with chronic pain histories ranging from nine months to 20+ years, were interviewed. Thematic saturation was reached after ten participants, with no new salient ideas being identified. Demographic details of participants are documented in Table 1. The participants’ experiences of chronic pain were wide ranging (Table 1). Experience of therapy that participants shared in interviews included a mix of occupational therapy and physiotherapy (Table 1).

**Table 1:** Participant demographics

Participant	Age	Gender	Duration of	Pain-related condition(s)/past	#Therapy discussed	*Past experience
(pseudonym)	(years)		chronic pain	medical history/comorbidities	in interview	of OT/PT
Campbell	46	Male	20+ years	Initiating condition not known, possibly work-related	OT	PT
Joe	65	Male	9 months	Brachial plexus injury 12 months prior to interview; nerve transfer 3 months prior to interview; torn rotator cuff	OT	PT
Pam	57	Female	13 months	Gluteal tendon tear; hip bursitis; neck and back injury	PT	None
Gina	71	Female	8 years	Bilateral knee replacements; Sjogren’s syndrome	PT	PT
Evie	38	Female	4 years	Knee surgery 4 years prior	PT	PT
Lena	65	Female	20+ years	Fibromyalgia; mast cell activation disorder; burst appendix 3 years prior to interview; osteoarthritic spine; breast cancer 18 months prior to interview	PT	PT
Thea	41	Female	20+ years	Grave’s disease; psoriatic arthritis with enthesitis; ankylosing spondylitis	PT/OT	PT/OT
Jenny	57	Female	18 months	Shoulder surgery (workplace injury); appendicectomy &bowel sepsis 14 months prior to interview; lymphedema	PT	PT
Mike	59	Male	4 years	Initiating condition not known; mental health conditions; depression	PT	PT
Gary	64	Male	2 years	Lower leg complex fracture; multiple orthopaedic and plastic surgeries	PT	PT

Right-hand columns indicate #the experience and type of therapy that was predominantly discussed during interview, and whether participants had *past experience of either occupational therapy or physical therapy that was not discussed explicitly during the interview.

To increase transferability of the findings, data are reported with rich descriptions [36] and relevant quotes are used to illustrate themes and sub-themes [39]. Participants’ names have been replaced with pseudonyms.

A total of six themes were identified in the participants’ narratives. Three of the themes that emerged were consistent with the findings of the Liddiard et al. [32] concept analysis of meaningfulness in chronic pain rehabilitation, namely 1) *personally valued*; 2) *self-defined*; and 3) *relevant to self-identity* (Table 2).

**Table 2:** Overview of themes and sub-themes

Theory -driven themes	Data driven themes
Personally valued	Genuine connection
Subthemes:	Subthemes:
•Holds value for me	•Authentic
•Individualized	•Not just a number
•Expectations – mine	•No judgement
•Expectations – the therapist’s	•Invested
•Progress or improvement	•Listens or hears me
•Engaged or enthused	•Comfort support and empathy
•Age and gender characteristics	•Friendly open and approachable
Self-defined	Credible
Subthemes:	Subthemes:
•Identified by me	•Knows chronic pain
•Empowered	•Professional knowledge or experience
	•Biopsychosocial perspective
	•Professional behaviour
	•Trust
	•Connects or refers to other professionals
Relevant to self-identity	A guiding partner
Subthemes:	Subthemes:
•They know me as a person	•Helps make things clear
•Relates to self-identity	•Where you’re at
•Brings you joy	•Teaches or explains
	•Coping mechanisms and practical strategies
	•Monitors and adjusts
	•Respectful partnership
	•Tuning me up

Theory-driven themes were previously identified through a concept analysis and upheld from the client perspective through coding and thematic analysis. Data-driven themes were not previously identified and emerged solely from the participants’ transcripts.

A further three new emergent themes were also identified: 4) *genuine connection*; 5) *credible*; and 6) a *guiding partner* (Table 2). In some instances, participants’ comments were reflective of more than one theme, and this was recorded as multiple codes.

The following three themes were previously identified in the rehabilitation literature from predominantly researcher/therapist perspectives, however, through the process of coding and thematic analysis the same themes were apparent from the participant perspective as well.

### 3.1 Personally valued

Participants believed that rehabilitation was more meaningful when it held *personal value*. They indicated that this may have an impact on client-engagement both at the time, and on completion of the therapy:

*If you cherish it, and value it, you*’*re gonna do it differently.* [Gary, line 251].

*At the end of the program they trusted me that I was going to go home and keep up with the program. Because I’d gotten a lot out of it.* [Mike, line 438].

Rehabilitation was more meaningful if the therapist had realistic expectations and respected what the client felt they could cope with:

 ...  *he was very patient* ...  *he was just lovely, and he would only push me as far as I could go.* [Jenny, line 233].

Some participants had strong expectations that they would achieve what they most valued, and that this served to drive them and add meaning to therapy:

*Ah, expectations* ...  *I will walk, I will. One way or the other. I might end up with a limp. And I*’*ll get on that bike.* [Gary, line 589].

In other instances, the therapy had meaning because it was structured around their personal interests:

*It*’*s not the exercise I go for* ...  *that*’*s what she impressed upon me, that it doesn*’*t matter what you*’*re doing, you*’*ve got to be loving it.* [Thea, line 394].

For rehabilitation to be meaningful, participants identified a need to see progress towards a *personally valued* goal, and found meaning in the therapist’s efforts to highlight this progress, or help them understand any lack of progress:

 ...  *remembering that the week before I was doing okay, this week I*’*m not doing so okay.* “*Okay, so what have you done this week? What have you been doing?*” [Jenny, line 410].

### 3.2 Self-defined

Participants’ descriptions of meaningful rehabilitation reflected their preference for working on *self-defined* goals with expert input from the therapist:

*I think ultimately you trust their professional judgement on which way to go, but just having a bit of choice in where it*’*s going, I think really helps.* [Pam, line 185].

*Making those goals* ...  *alongside her, I suppose, as opposed to them saying,* “*there you go, we*’*re the experts*”. [Thea, line 278].

The opportunity to define their own goals was described as meaningful by some participants because it empowered them:

 ...  *she wants to empower her patients* ...  *she doesn*’*t want them to come back twice a week every week...she wants* ...  *them to...be in charge of their own rehab.* [Thea, line 367].

### 3.3 Relevant to self-identity

Participants stated rehabilitation was more meaningful when their therapist knew them as a person, and, as a result, they perceived that aspects of therapy were tailored to their *self-identity*:

*It’s gotta be tailormade for the individual. Right? And as far as I’m concerned, the way I look at it is, he has done that. He has looked at me, got to know me, understood you know?* [Gary, line 533].

This ‘tailoring’ by the therapist considered aspects of the person such as roles, values and past meaningful activities, and demonstrated the therapist’s knowledge of their client’s character and sense of *self-identity*:

*So, long term* ...  *the idea was that I would walk again and ride my motorbike again. That was my long-term goal. That is what*’*s kept me driving all along.* [Gary, line 320].

Participants explained that when they were encouraged to do more of the things that brought personal joy and a sense of meaning in life, the rehabilitation was more meaningful:

*She*’*s like yeah well, you*’*ve still got your walk in, but you had it in a better way because you were where you wanted to be, you*’*re at the beach, you were with a girlfriend.* [Thea, line 467].

In addition to the themes above, three more themes not previously represented in the literature also emerged.

### 3.4 Genuine connection

Participants described a *genuine connection* with their therapist in, what they perceived as, a reciprocal and authentic relationship. Some participants explained that at times where they had to accept negative feedback, or an unexpected lack of progress, the *genuine connection* experienced with their therapist helped them maintain a sense that their rehabilitation was meaningful. This *genuine connection* relied significantly on a therapist who displayed characteristics of compassion, for example empathy, openness, approachability, a sense of humor, and the ability to make a participant feel heard and validated:

*So, when you*’*re spoken to compassionately and you*’*re told the truth in a compassionate way, then that*’*s okay. [Jenny, line 549]*.

 ...  *Adam is the only one* ...  *I feel like actually really heard me in the few sessions that I went to. [Pam, line 163].*

The authentic nature of the client’s connection with the therapist emerged as a key feature of this *genuine connection*. When participants felt like a commercial proposition, or just a number, they described this as non-meaningful.

*Yeah, so it*’*s very important that my therapist makes [me] feel like I*’*m the only one there at that moment. I*’*m not just another slot on the diary.* [Jenny, line 396].

By contrast a meaningful experience included a *genuine connection*, where participants found the therapist to be warm, and personal, with reciprocal emotional interactions bridging the therapist/client gap:

*And Carlo was one of those who treated you, but he also treated you personally as well. So, it soothes the soul.* [Gary, line 207].

Participants’ comments reflected a belief that their therapist would know they had done everything possible to follow the therapist’s recommendations, and would not judge them when chronic pain or fatigue reduced their ability to engage in therapy:

*He was very kind at the time, and he said, you know, you*’*ve done everything you can do.* [Gina, line 235].

### 3.5 Credible

From the participants’ descriptions of both meaningful and non-meaningful rehabilitation, a key theme was a perception that when the therapist was *credible* the experience was more meaningful. The Macquarie Dictionary defines the adjective ‘credible’ as “capable of being believed; believable” and “worthy of belief or confidence; trustworthy” [41]. According to participants, this credibility was conveyed in different ways. One unsurprising finding was that participants described greater confidence and trust when the therapist’s behavior demonstrated their experience, knowledge and professional skill in chronic pain:

 ...  *they shared [their] knowledge and experience. And that was meaningful.* [Mike, line 432].

In addition, however, some less obvious behaviors also led to a perception of credibility for participants. For example, when the therapist was confident enough in their own ability that they were willing to admit when they were unable to help:

*If they admit that they don*’*t know and they can*’*t do much to help, I always find that is frustrating, but I would find them being honest better than the other way, and saying,* “*come and see me every week for six years and we*’*ll see what we do*”. [Lena, line 276].

or when they were willing to refer to other relevant chronic pain health professionals:

 ...  *help with giving you a group of people if necessary, like if they think you need to see a different type of person* – *a different type of therapist, I think that would be really wonderful.* [Lena, line 359].

Credibility came not just from the therapist having training or extra knowledge of chronic pain in an academic sense, but also from the therapist knowing chronic pain as a lived experience – either their own, or through close connection with former clients, family or friends with chronic pain.

*He’s a chronic pain patient himself* ...  *so he’s someone who’s been through it, who lives through it.* [Evie, line 191].

Participants perceived that when the therapist knew the lived experience of chronic pain, they behaved sympathetically and allowed for the participant’s chronic pain challenges and barriers. For example, participants described the experience to be more meaningful when the therapist accepted that they may be less engaged on a day when pain or fatigue was particularly debilitating:

*Yeah, it’s not just a case of saying* “*you*’*re being weak, suck it up*” *but it was,* “*no we understand*”. [Mike, line 93].

*I think you just built a much better rapport with the therapist if they did understand how chronic pain, and the fatigue and everything, affects you. I think for most people they just think, sounds like a bit of bullshit.* [Jenny, line 347].

Knowing chronic pain in this way, participants perceived the therapist was better able to relate to variations in their client’s experience, which made the therapist more credible and rehabilitation more meaningful:

*Yeah, I think it*’*s probably the most important thing is to say* “*where are you today?*”*, because you could be on level 9, or you could be on level 3, and how you*’*re going to relate to me when I*’*m on level 9 is different to when I*’*m at level 3.* [Campbell, line 449].

### 3.6 A guiding partner

Participants described an encounter to be more meaningful when the therapist acted as a *guiding partner* in their chronic pain rehabilitation. Some explained that they were guided to change their future direction because their therapist offered an alternate perspective which allowed them to reflect on their situation and determine how they would like to change:

*One of the things they actually did was video record us [on Day 1]* ...  *walking to a chair sitting down and standing up and then walking back. I look like a 90-year-old man on a walking stick* ...  *I felt very sad for myself, and I thought that’s not me* ...  *I’ve gotta get better...* [Mike, line 191].

Rehabilitation was more meaningful for some participants when their therapist would check ‘where they were at’, either in relation to what they could physically handle that day, or their current mental and emotional reserve to deal with pain or other demands. They found rehabilitation meaningful when the therapist was able to ‘meet’ them ‘where they were at’, even if there was no forward momentum in their rehabilitation at that point:

 ...  *that*’*s what we*’*re doing with Susan. She*’*s going* “*where are you, and what are you doing; do you need a massage today or would you want to talk?*” [Campbell, line 427].

If the therapist was able to guide them on aspects of the chronic pain experience, some participants described a sense of being empowered to cope on their own, which they felt was more meaningful. Their descriptions included both a sense of being empowered and that their therapist had confidence in their ability:

*Then when he’s not there* ...  *I would say,* “*well what would Carlo do? What would Carlo say?*”*. All right? And it gave me the encouragement then to go forward from where I am now, you know, without him, being able to do what I can.* [Gary, line 126].

Participants perceived their *guiding partner* to be a meaningful resource to help develop coping mechanisms and strategies. At the same time, the therapist monitored, adjusted and personalized the direction, or trajectory, of their rehabilitation:

 ...  *one of the happiest things in my life is to go bowling with my granddaughter and I can*’*t do that now. So, I would discuss something like that about* “*how am I going to get from this point now to that point again?*” *And so, it was important* ...  *to be able to talk about me personally in that respect.* [Jenny, line 527].

Some participants found it meaningful that their therapist, as a *guiding partner*, focused on living well with chronic pain rather than taking a curative/fixing lens for the pain interventions, and this extended to everyday activities:

*Do the* ...  *shopping [online], and then take that energy you would have had to use and go to the beach and have a coffee. She helped me see that I could switch things around.* [Thea, line 478].

Participants related an experience of now living well with chronic pain but, on occasion, also returning to their therapist to seek more guidance as a ‘tune up’, which indicates that this engagement with the therapist was meaningful to them:

*We need tuning up, and that*’*s what my OT is now, my tune up.* [Campbell, line 373].∥

## 4 Discussion

This study explores the experience of meaningfulness in rehabilitation from the perspective of people with chronic pain. Although there is a paucity of research in this area, client-defined meaningfulness is important because of the strong influence it can exert on client engagement in therapy. The findings from this study demonstrate that clients describe a meaningful rehabilitation experience as having a *genuine connection* with a *credible* therapist, who can act as a *guiding partner* to address what the client *self-defines* as *personally valued*, and relevant to their *self-identity*.

The participants in this study offered new insight into what makes rehabilitation meaningful. Of the themes that emerged from participants’ interviews, three aligned with existing themes identified in the 2019 concept analysis [32] which predominantly represented the views of therapists and researchers from rehabilitation literature. These were *personally valued*; *self-defined*; and relevant to their sense of *self-identity*. However, three further themes, that had not previously been identified, became apparent through the perspective offered by people with chronic pain, these being *genuine connection; credible*; and *guiding partner*. This appears to indicate that the current literature has not fully captured the construct of meaningfulness in chronic pain rehabilitation from the client’s perspective and underscores the value of this study to inform future research. The findings from this study are supported in recent literature, however, they also encourage further examination of some assumptions about chronic pain rehabilitation. In particular, the themes of *genuine connection*, *credible* and *guiding partner* offer valuable insight into the client perspective of the experience. The following will explore these themes in greater depth.

### 4.1 A genuine connection

The theme of a *genuine connection* between therapist and client warrants further examination. Participants described a more meaningful rehabilitation encounter where the therapist was compassionate, authentic and non-judgmental, which encourages a reappraisal of the concept of professional boundaries. Biomedical models taught over past decades suggest that therapists should “bracket” their own experiences in order to forefront those of their client [42]. Some therapists may feel that they should establish professional boundaries and create a barrier to hold their own personal interests and experiences back from the therapeutic relationship [42]. The *genuine connection* described by our participants suggests that a reciprocal and authentic relationship with their therapist was highly meaningful. They found meaning in an opportunity to give of their own knowledge and experience, while at the same time benefiting from the knowledge and experience of their therapist. It is not clear that all rehabilitation training programs prepare new therapists for this type of relationship.

These findings have important implications for current therapists and for education programs that prepare future therapists. The professional-client boundary has been an ethical discussion over many decades. It remains a divisive topic [43] and is difficult to teach in a nuanced manner to healthcare professionals [44]. Therapists, whose training and practice aligns with assuming the traditional role of ‘objective expert’ [45], may have difficulty learning to negotiate this boundary line, while offering clients a meaningful rehabilitation experience. The concept of the professional-client boundary may warrant further investigation in light of this emergent information that focuses on the mostly overlooked perspective of clients, who describe a *genuine connection* with their therapist as part of a meaningful rehabilitation experience.

The *genuine connection* described as meaningful by these participants, may also encourage therapists to reflect on the concept of client-centeredness, a long-accepted tenet of rehabilitation where the person and their own goals are the focus of the therapy. It might be assumed that this focus is sufficient to ensure a client is fully satisfied with their rehabilitation, however, the *genuine connection* described by participants suggests that a meaningful encounter goes further than simply centring therapy on the client. The language and practice of relationship-focused therapy, as one element of a client-centered approach, is becoming more visible in rehabilitation literature [42]. Participants in this study described an intentional relationship with their therapist, and rather than expecting the full focus would be on them as the client, they explained that a reciprocal human connection was meaningful. Framed in this manner, therapists should be open to an authentic human connection, and be prepared to reflect on the nature of their therapeutic relationship as it evolves. A *genuine connection* may offer the therapist a valuable opportunity to understand the lived experience of chronic pain from their clients’ perspectives. It is plausible that this, in turn, could contribute to the view that future clients have of them as a *credible* chronic pain health professional.

### 4.2 The credible therapist

Participants perceived a *credible* therapist to provide a more meaningful rehabilitation encounter. Subthemes that contributed to this theme of *credibility* included that the therapist displayed professional knowledge, and behavior, along with a biopsychosocial perspective (Table 2). This is perhaps an unsurprising finding given that therapists themselves have identified a need for greater chronic pain training, in particular related to psychosocial knowledge and skills [25, 26]. In another subtheme, participants expressed that the experience was more meaningful when the therapist ‘knew chronic pain’ at a deeper level. The implication appeared to be that the therapist would not simply be trained in chronic pain skills, knowing chronic pain in an academic sense, rather they would know chronic pain from lived experience, either their own, or past clients and family or friends with whom they had sufficient connection to gain this richer perspective. Given the finding that rehabilitation is perceived to be more meaningful when a therapist develops a *genuine connection* with their client, it may be that those clients then privilege the therapist with their rich perspective of the lived experience of chronic pain.

This raises interesting questions about the nature of the chronic pain training that therapists need to be exposed to. It is not clear whether evidence has previously highlighted the importance of this type of *credibility*, based on truly knowing chronic pain, to create a more meaningful rehabilitation experience. This finding also highlights that further research is needed into how this knowledge can best be translated into practice. For example, is it possible to convey lived experience of people with chronic pain through continuing professional development and university training, or do therapists only truly gain this rich understanding through experience?

### 4.3 The role of guiding partner

In contrast to traditional practice, where a therapist takes responsibility to provide expert guidance [46], participants strongly endorsed a greater experience of meaningfulness when the therapist assumed the role of *guiding partner*. People with chronic pain often feel disconnected from the activities and relationships that give them a sense of purpose [47]. They may seek to re-establish meaningful activities with support from a therapist whom they trust, and who gives them confidence to decide their own direction [48]. This is greater than just taking the role of teacher or expert. Over the past two decades there has been a focus on therapists teaching people about chronic pain through educational psychology principles [49]. This growing direction has raised awareness amongst therapists about the importance of a biopsychosocial approach and encouraged a move away from an outdated biomedical perspective of pain [50]. However, it is important that this educational psychology approach is paired with other skills to reduce the risk that therapists adopt a role of teacher-expert rather than *guiding partner*.

Participants described the *guiding partner* therapist as someone who would offer an alternate perspective for them to reflect on, so they could adjust their vision of their future direction. The *guiding partner* was also someone who would ‘check-in’ with them, in a similar way to psychological approaches that require the therapist to be present but not always actively intervening [51]. The *guiding partner* that participants described was also someone who could help them to develop coping mechanisms and strategies to self-manage their pain experience. These descriptions are as much about facilitating clients’ self-efficacy, as they are about educating them on practical strategies. It is important that as therapists begin to work with people who have chronic pain, equal value is placed on the skills they need to translate pain knowledge for their clients, and those that will enable them to fulfill the role of *guiding partner*.

As a *guiding partner* participants discussed the ability of the therapist to monitor, adjust and personalize the trajectory of their ‘journey’ towards living well with chronic pain. Assuming a role of *guiding partner* rather than teacher-expert, however, may require an active commitment from therapists to reflect on the role they personally tend towards in their own practice. For some therapists, additional training may be required to assume the role of *guiding partner* and the challenges it presents for negotiating and communicating clear co-authored goals.

### 4.4 Guiding partner, genuine connection and the power differential

For the therapist to fulfill a role as *guiding partner* they must accept that power is shared between therapist and client. The power differential in a therapeutic relationship is something that therapists are increasingly encouraged to consider, and it has received growing attention in the literature [52]. The environment that a new therapist enters into following university may impact on their early development of professional values through a process of professional socialization [53]. If a therapist commences their career in an organization or institution where traditional or biomedical approaches are valued, they may lose sight of the need to examine the power differential, regardless of their training in these concepts. If they later move into the chronic pain field, it may be relevant to once again reflect on their own values and revisit the concept of power in their therapeutic relationships. This is especially important in light of the compassionate, authentic and non-judgmental *genuine connection* that people with chronic pain find meaningful.

A relationship of shared power has obvious benefits. One may be that the client feels empowered to not only ask the therapist for advice, but also to ask the therapist to share what they themselves would do if they were in the same situation [54]. This honest discussion may allow the client to benefit from the therapist’s contemporary pain knowledge and opinion, while at the same time retaining the control and autonomy in their own rehabilitation [54]. While the *genuine connection* with a *credible* therapist as *guiding partner* was described as meaningful by people with chronic pain in this study, it may also contribute to better outcomes. Therefore, further research is needed to examine exactly what the *genuine connection, credibility* and *guiding partnership* encompass, and how therapists may enact these in a pragmatic way given the requirements for accountability and time pressure that the health system currently imposes on rehabilitation.

### 4.5 Limitations

As with all studies there were limitations. The researcher brings a specific socio-cultural lens to the analysis; however, this was mitigated by using a range of well-established methodological strategies to ensure study trustworthiness. Some participants had past histories with rehabilitation (Table 1) and while longevity of the therapeutic relationship and perceived meaningfulness have not been linked in the literature, there is potential that this had an impact on findings, and this will be an important question for future research. The scope of this study focused on what people with chronic pain perceived to be meaningful in the rehabilitation encounter. Further research is needed; for example, what criteria do clients use to determine therapist *credibility*? How can elements of meaningful rehabilitation, such as the *genuine connection,* be achieved within the current constraints of many healthcare systems? What are potential negative outcomes of shared decision-making in the *guiding partner* role?

## 5 Conclusions

This study explored the gap in the evidence-base regarding the experience of meaningfulness in rehabilitation from the perspective of people with chronic pain. Participants in this study describe meaningful rehabilitation to include the experience of a *genuine connection*, with a *credible* therapist, who can act as a *guiding partner* to address what the client *self-defines* as *personally valued* and relevant to their *self-identity*. Current educational psychology approaches adopted by therapists in chronic pain management should be paired with other therapeutic skills and attitudes required for *genuine connection* and *guiding partnership* approaches. This would reduce the risk that therapists adopt a position of teacher-expert along with the inherent power differential that this sets up. In some practice environments, therapists are encouraged to maintain professional boundaries; however, based on the results of this study, people with chronic pain find rehabilitation more meaningful when they have a *genuine connection* with their therapist. This suggests there are complex and potentially competing requirements in rehabilitation service provision, and more nuanced examination is clearly needed. The experience of meaningfulness in rehabilitation for people with chronic pain examined in this research warrants further study. Therapist education and skills to facilitate client engagement and relevant outcomes for clients are important directions for future research. This study lays a foundation to address existing gaps in the evidence-base concerning client-identified meaningfulness in the rehabilitation encounter for people with chronic pain.

## Author contributions

KL, AR and CB designed the study. KL carried out the data collection and led the data analysis with guidance from AR and CB. HDJ carried out the independent audit of data. All authors contributed to writing the manuscript and have approved the final article.

## Ethical approval

Approval was granted by the Edith Cowan University Human Research Ethics Committee (approval no. 21008; 20/11/2019) prior to recruitment. Informed consent was obtained from all participants on the basis that published responses would be anonymous. Pseudonyms have been used throughout. This study was conducted in accordance with the Declaration of Helsinki of 1964.

## Conflict of interest

The authors declare that the research was conducted in the absence of any commercial or financial relationships that could be construed as a potential conflict of interest.
